# Characterization of resin extracted from guayule (*Parthenium argentatum*): A dataset including GC–MS and FT-ICR MS

**DOI:** 10.1016/j.dib.2020.105989

**Published:** 2020-07-05

**Authors:** Mostafa Dehghanizadeh, Feng Cheng, Jacqueline M. Jarvis, F. Omar Holguin, Catherine E. Brewer

**Affiliations:** a Department of Chemical and Materials Engineering, New Mexico State University, P.O. Box 30001 MSC 3805, Las Cruces, NM 88003, USA; b Department of Plant and Environmental Sciences, New Mexico State University, Las Cruces, NM 88003, USA

**Keywords:** Guayule, *Parthenium argentatum*, Natural resin, Mass spectroscopy, Complex mixtures, Terpenes

## Abstract

Guayule (*Parthenium argentatum*), a shrub native to the arid region of the U.S. southwest and Mexico belonging to the Asteraceae family, is a source of high quality, hypoallergenic natural rubber with applications in pharmaceutical, tire, and food industries. Production of rubber results in a substantial amount of resin-containing residues which contain a wide variety of secondary metabolites (sesquiterpene esters, triterpene alcohols, fatty acids, etc.). In order to enhance the economic viability of guayule as an industrial crop, value-added use of the residues is needed and has the potential to reduce gross rubber production costs. The main objective of this research is the characterization of guayule resin using rapid and accurate analytical techniques to identify compounds of potential commercial value. Guayule resin is inherently complex and includes many high-molecular-weight and non-volatile compounds that are not easy to observe using traditional chromatographic techniques. The combination of two mass spectroscopy techniques: gas chromatography mass spectroscopy (GC–MS) and high-resolution Fourier transform ion cyclotron resonance mass spectroscopy (FT-ICR MS), were used to characterize the composition of the extracted resin from guayule (*Parthenium argentatum*). FT-ICR MS was used to characterize hundreds of compounds with over a wide range of molecular weights and degrees of aromaticity at higher levels of mass accuracy than other forms of mass spectrometry. GC–MS was used to identify volatile compounds like mono- and sesquiterpene compounds.

**Specifications Table**

**Table:** 

**Subject**	Chemical engineering (General), Analytical chemistry
**Specific subject area**	Characterization of resin from biomass
**Type of data**	Tables Figures
**How data were acquired**	Gas chromatography - mass spectroscopy (GC–MS): 7890A, Agilent Technologies. Libraries: ADAMS and NIST MS Search 2.0 Fourier transform ion cyclotron resonance mass spectroscopy (FT-ICR-MS): 9.4 T FT-ICR mass spectrometer. Modular ICR data acquisition system (PREDATOR) for data collection. PetroOrg software for generating mass spectral lists.
**Data format**	Raw Analyzed Filtered
**Parameters for data collection**	GC–MS: DB-5 MS capillary column (30 m × 0.25 mm, 0.25 mm, Agilent Technologies) Solvent: carbon disulphide The oven program: initial temperature 50 °C, 1 min isothermal, 3 °C/min ramp to 320 °C, 10 min isothermal Ion source: EI Ion source temperature: 250 °C FT- ICR MS: Solvent: toluene Capillary rate: 50 μL/min. Sheath gas: nitrogen (60 psi) Nebulization gas: nitrogen Photon source: krypton VUV lamp Dopant: toluene Time domain signal acquisition period: 4.1s
**Description of data collection**	Resin from commercial pilot-scale bulk rubber extraction of guayule (*Parthenium argentatum*) was characterized by GC–MS and FT-ICR MS without any additional separation or pretreatment. GC–MS used retention times and matches to compound databases to identify volatile compounds. FT-ICR-MS used high resolution mass to charge ratios to provide molecular formulae, carbon number, and double bond equivalents for ionizable compounds, including many not detectable by GC–MS. Composition is based on relative abundance.
**Data source location**	Institution: New Mexico State University and National High Magnetic Field Laboratory City/Town/Region: Las Cruces, NM and Tallahassee, FL Country: USA
**Data accessibility**	With the article
**Related research article**	Author's name: Feng Cheng, Mostafa Dehghanizadeh, Meshack A. Audu, Jacqueline M. Jarvis, F. Omar Holguin, Catherine E Brewer Title: Characterization and evaluation of guayule biomass and processing residues as potential feedstock for biofuel and chemical production Journal: *Industrial Crops & Products* DOI: 10.1016/j.indcrop.2020.112311

**Value of the Data**

• This dataset represents a comprehensive characterization of guayule resin using several complementary analysis methods.
• Researchers working on natural resin characterization, processing, and utilization can benefit from this data.
• Quantitative and qualitative composition data for guayule resin can be used to select future separation techniques for analysis and to identify potential applications of separated resin fractions based on their expected compositions.
• Data from GC–MS is commonly available for resin samples and provides a common basis for comparison between this data and previously collected data for other resin samples.
• The FT-ICR MS data provides information about a wider range of molecular weights than GC–MS data due to the wider range of molecules that can be detected using the ionization method.

## 1 Data

**Fig. 1:**
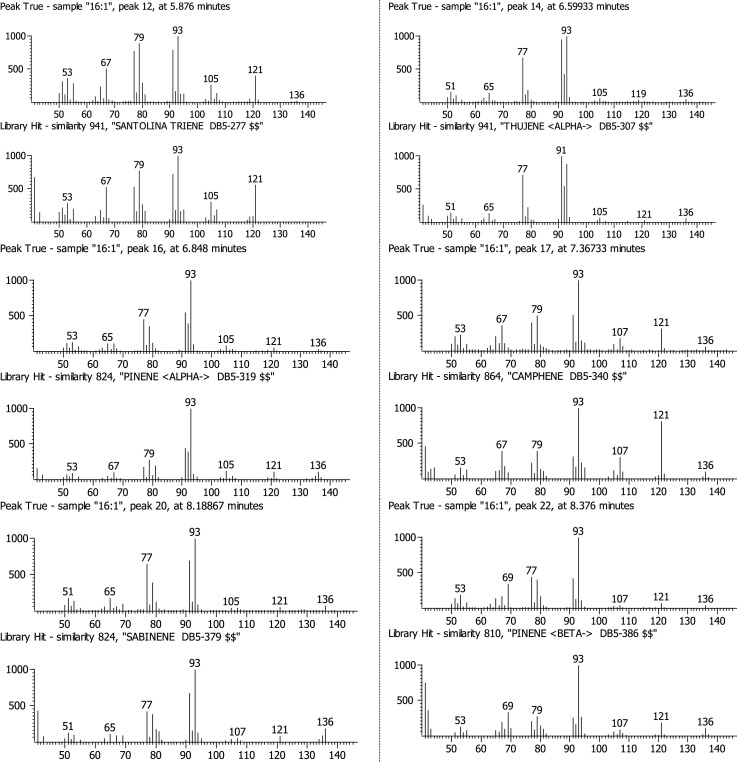
Measured GC-MS spectra (top of each pair) and the corresponding library spectra (bottom of each pair) of the most abundant compounds.

**Fig. 2:**
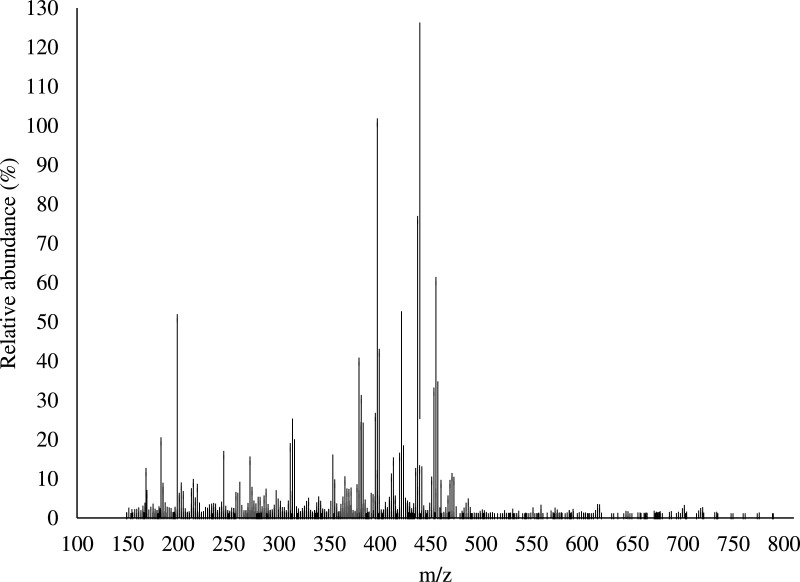
Broadband positive-ion APPI FT-ICR MS for resin from guayule (*Parthenium argentatum*).

Data here includes: a table of terpene molecules identified within the guayule resin sample by GC–MS with the parameters used for identification (Table 1); a table of compounds identified by negative-ion APPI FT-ICR MS of guayule resin with the mass-to-charge ratios and assigned molecular formulas for each compound (Table 2); a table of compounds identified by positive-ion APPI FT-ICR MS of guayule resin with the mass-to-charge ratios and assigned molecular formulas for hydrocarbon-containing compounds with >5% relative abundance (Table 3); a figure showing the experimentally collected GC mass spectra (top) and library mass spectra (bottom) for the terpene compounds listed in Table 1 (Fig. 1); a figure showing the broadband positive-ion APPI FT-ICR MS spectrum of guayule resin corresponding to compounds in Table 3 (Fig. 2), a figure of color-coded isoabundance plots of the compounds in the hydrocarbon (HC) and oxygenated molecule classes from positive-ion APPI FT-ICR MS of guayule resin corresponding to the compounds in Table 3 and Fig. 2; and a figure of carbon number distribution derived from negative-ion APPI FT-ICR MS (Fig. 4). A broadband negative-ion APPI FT-ICR MS spectrum, heteroatom class distributions data by positive- and negative-ion APPI FT-ICR MS, and a color-coded isoabundance contour plots for the hydrocarbon (HC), N-, and O-containing heteroatom classes from negative-ion APPI FT-ICR MS have been reported in Figs. 5, 6, and 7 of the original research article, respectively [1].

**Table 1:** Identified possible terpenes in guayule resin by GC-MS with similarity, retention time, signal/noise ratio (S/N), peak area, difference between calculated and observed KI.

Terpene	Similarity	RetentionTime (min)	S/N	Area	KI	Calculated KI	Error%
santolina triene	941	5.87667	877.6	853,685	909	919.3	1.13
alpha-thujene	941	6.59933	283.3	308,322	930	936.3	0.68
alpha-pinene	824	6.84867	9092.8	10,100,415	939	942.2	0.34
camphene	864	7.36733	274.66	802,667	954	954.4	0.04
thuja-2,4(10)-diene	946	7.50867	661.47	1,184,850	960	957.7	0.24
sabinene	824	8.18867	215.91	817,092	975	973.8	0.13
beta-pinene	810	8.376	17,359	51,598,177	979	978.2	0.08
myrcene	852	8.82467	2283.5	6,337,568	990	988.8	0.13
alpha-phellandrene	881	9.43067	171.22	484,042	1002	1003.0	0.10
o-cymene	922	10.17	528.25	881,908	1026	1020.5	0.54
limonene	888	10.3673	2472.2	12,104,532	1029	1025.1	0.38
beta-phellandrene	879	10.3947	23.137	236,992	1029	1025.8	0.31
beta-ocimene	913	11.1013	103.69	303,186	1037	1042.4	0.52
terpinolene	782	12.7147	60.831	175,948	1088	1080.5	0.69
cis-pinocarveol	903	15.1293	353.31	1,901,222	1139	1137.4	0.14
trans-pinocamphone	840	15.992	155.56	350,926	1162	1157.7	0.37
lavandulol	852	16.266	116.44	341,186	1169	1164.2	0.41
borneol	864	16.538	339.89	432,096	1169	1170.6	0.14
thuj-3-en-10-al	900	17.5667	220.53	1,514,848	1184	1194.8	0.92
verbenone	891	18.1207	406.23	2,663,266	1205	1207.9	0.24
carvone	862	19.7707	74.98	107,019	1243	1246.8	0.31
phellandral	806	21.234	78.368	457,908	1275	1281.3	0.49
lavandulyl acetate	931	21.662	629.69	2,100,790	1290	1291.4	0.11
delta-elemene	801	23.6113	418.91	2,986,082	1338	1337.3	0.05
alpha-cubebene	903	24.2713	484.23	2,363,147	1351	1352.9	0.14
beta-maaliene	878	25.6667	770.08	5,997,644	1382	1385.8	0.27
beta-elemene	916	26.1027	488.82	3,734,545	1390	1396.1	0.44
alpha-gurjunene	839	26.7747	231.96	1,414,908	1409	1411.9	0.21
beta-isocomene	864	26.8713	661.54	8,080,396	1408	1414.2	0.44
(E)-caryophyllene	893	27.328	4594.4	56,916,328	1419	1425.0	0.42
beta-copaene	892	27.708	232	1,279,844	1431	1433.9	0.20
alpha-guaiene	843	28.0667	457.33	8,720,186	1439	1442.3	0.23
alpha-humulene	881	28.7593	1157.1	7,226,074	1455	1458.7	0.25
allo-aromadendrene	885	28.9487	296.67	2,297,528	1460	1463.2	0.22
ar-curcumene	927	29.9347	707.96	2,801,211	1480	1486.4	0.43
bicyclogermacrene	902	30.43	1173.5	8,706,955	1500	1498.1	0.13
gamma-cadinene	900	31.172	2159.7	19,008,266	1514	1515.6	0.10
delta-cadinene	891	31.4727	5321.3	81,078,904	1523	1522.7	0.02
alpha-cadinene	819	32.0853	669.48	2,890,705	1538	1537.1	0.06
alpha-calacorene	843	32.2433	1279.2	3,090,123	1545	1540.8	0.27
elemol	867	32.8433	9640.4	263,133,915	1549	1555.0	0.39
(Z)-nerolidol	805	33.278	3574.8	33,729,371	1563	1565.2	0.14
globulol	890	34.032	126.3	5,048,402	1590	1583.0	0.44
beta-oplopenone	807	34.6953	124.57	700,252	1607	1598.6	0.52
gamma- eudesmol	833	36.2067	1509.4	69,688,360	1632	1630.9	0.07
caryophylla-4(14),8(15)-dien-5a-ol	937	36.2667	1308.3	5,850,574	1640	1635.7	0.26
beta- eudesmol	841	37.044	1509.4	69,688,360	1632	1630.9	0.1
eudesma-4(15),7‑dien-l-beta-ol	843	38.044	161.2	2,482,762	1686	1677.6	0.50

**Table 2:** Identified compounds with > 5% relative abundance in guayule resin by negative-ion APPI FT-ICR MS. DBE, double bond equivalent.

No.	Relative abundance (%)	Experimental m/z	Error^a^ (ppm)	Molecular formula	DBE
1	100	473.4	0.0077	C_30_H_50_O_4_	6
2	99.7	471.3	−0.0137	C_30_H_48_O_4_	7
3	88.0	408.3	−0.0402	C_27_H_36_O_3_	10
4	67.2	469.3	0.0075	C_30_H_46_O_4_	8
5	56.4	455.4	−0.0462	C_30_H_48_O_3_	7
6	48.8	279.2	0.0137	C_18_H_32_O_2_	3
7	38.7	483.3	−0.0454	C_30_H_44_O_5_	9
8	30.0	485.3	−0.0451	C_30_H_46_O_5_	8
9	29.0	498.3	−0.0047	C_30_H_42_O_6_	10
10	27.0	255.2	0.0149	C_16_H_32_O_2_	1
11	26.2	379.3	−0.016	C_26_H_36_O_2_	9
12	25.2	453.3	−0.0245	C_30_H_46_O_3_	8
13	25.2	277.2	0.0135	C_18_H_30_O_2_	4
14	23.4	406.3	−0.0405	C_27_H_34_O_3_	11
15	22.7	499.3	−0.0147	C_30_H_44_O_6_	9
16	21.4	481.3	−0.0457	C_30_H_42_O_5_	10
17	21.0	487.3	−0.0448	C_30_H_48_O_5_	7
18	19.9	295.2	−0.0053	C_18_H_32_O_3_	3
19	19.8	293.2	0.0285	C_18_H_30_O_3_	4
20	16.7	367.4	−0.0427	C_24_H_48_O_2_	1
21	15.3	467.3	0.0074	C_30_H_44_O_4_	9
22	14.8	501.3	−0.0145	C_30_H_46_O_6_	8
23	13.9	395.4	−0.014	C_26_H_52_O_2_	1
24	13.6	283.3	0.0492	C_18_H_36_O_2_	1
25	12.9	448.3	−0.0363	C_30_H_40_O_3_	11
26	12.9	449.3	−0.0028	C_30_H_42_O_3_	10
27	12.8	514.3	−0.0345	C_30_H_42_O_7_	10
28	11.9	451.3	−0.0248	C_30_H_44_O_3_	9
29	11.7	393.2	−0.0293	C_26_H_34_O_3_	10
30	10.9	397.3	−0.0148	C_27_H_42_O_2_	7
31	10.6	391.2	−0.004	C_26_H_32_O_3_	11
32	10.5	515.3	−0.0052	C_30_H_44_O_7_	9
33	10.3	377.2	−0.0162	C_26_H_34_O_2_	10
34	10.0	388.2	−0.003	C_27_H_32_O_2_	12
35	9.8	361.2	0.0104	C_25_H_30_O_2_	11
36	9.6	281.2	0.0493	C_18_H_34_O_2_	2
37	8.6	339.3	0.0418	C_22_H_44_O_2_	1
38	8.6	453.3	−0.0146	C_29_H_42_O_4_	9
39	8.5	391.3	−0.0155	C_27_H_36_O_2_	10
40	8.4	403.2	−0.0287	C_27_H_32_O_3_	12
41	8.4	389.2	−0.0042	C_26_H_30_O_3_	12
42	8.3	496.3	0.0153	C_30_H_40_O_6_	11
43	8.2	465.3	−0.0142	C_30_H_42_O_4_	10
44	8.1	335.2	−0.0188	^C^_23_H_28_O_2_	10
45	8.0	433.3	−0.0263	C_29_H_38_O_3_	11
46	8.0	479.3	0.0165	C_30_H_40_O_5_	11
47	8.0	353.2	0.0393	C_24_H_34_O_2_	8
48	7.7	409.3	−0.0522	C_27_H_38_O_3_	9
49	7.4	435.3	−0.026	C_29_H_40_O_3_	10
50	7.4	387.2	−0.0044	C_26_H_28_O_3_	13
51	7.3	469.3	0.017	C_29_H_42_O_5_	9
52	7.3	359.2	0.0381	C_25_H_28_O_2_	12
53	7.3	467.3	0.0384	C_29_H_40_O_5_	10
54	7.2	463.3	0.0071	C_30_H_40_O_4_	11
55	7.2	423.3	−0.0161	C_27_H_36_O_4_	10
56	7.1	408.2	−0.0537	C_26_H_32_O_4_	11
57	7.0	363.2	−0.017	C_25_H_32_O_2_	10
58	7.0	557.5	0.0072	C_36_H_62_O_4_	6
59	7.0	451.3	−0.0148	C_29_H_40_O_4_	10
60	6.8	421.2	−0.0163	C_27_H_34_O_4_	11
61	6.8	395.3	−0.0036	C_26_H_36_O_3_	9
62	6.7	503.3	0.0453	C_30_H_48_O_6_	7
63	6.7	369.2	−0.0582	C_24_H_34_O_3_	8
64	6.7	489.4	−0.0036	C_30_H_50_O_5_	6
65	6.6	425.3	0.0076	C_27_H_38_O_4_	9
66	6.5	381.2	0.0078	C_24_H_30_O_4_	10
67	6.4	483.3	0.0053	C_29_H_40_O_6_	10
68	6.4	291.2	0.0285	C_18_H_28_O_3_	5
69	6.4	517.3	−0.0244	C_30_H_46_O_7_	8
70	6.3	512.3	0.0238	C_30_H_40_O_7_	11
71	6.3	311.3	−0.019	C_20_H_40_O_2_	1
72	6.2	333.2	−0.0191	C_23_H_26_O_2_	11
73	6.0	389.2	0.01	C_27_H_34_O_2_	11
74	5.8	373.2	−0.0435	C_26_H_30_O_2_	12
75	5.7	485.3	0.0054	C_29_H_42_O_6_	9
76	5.6	347.2	0.0106	C_24_H_28_O_2_	11
77	5.4	437.2	−0.0509	C_27_H_34_O_5_	11
78	5.3	449.3	−0.015	C_29_H_38_O_4_	11
79	5.3	409.2	−0.0657	C_26_H_34_O_4_	10
80	5.3	381.2	−0.0039	C_25_H_34_O_3_	9
81	5.2	375.2	−0.0165	C_26_H_32_O_2_	11
82	5.0	357.2	0.0381	C_25_H_26_O_2_	13
83	5.0	437.3	−0.0486	C_29_H_42_O_3_	9

a Error (*m/z*)= difference between theoretical and observed mass.

**Table 3:** Identified compounds in HC class with > 5% relative abundance in guayule resin by positive-ion APPI FT-ICR MS.

No.	Relative abundance (%)	Exp. m/z	Error^a^ (ppm)	Molecular Formula	DBE
1	15.6	183.1	−0.0169	C_14_H_14_	8
2	9.40	168.2	0.0130	C_12_H_24_	1
3	8.95	397.4	−0.0302	C_29_H_48_	6
4	6.39	203.2	−0.0140	C_15_H_22_	5
5	5.57	185.1	−0.0164	C_14_H_16_	7

a Error (m/z)= difference between theoretical and observed mass.

**Fig. 3:**
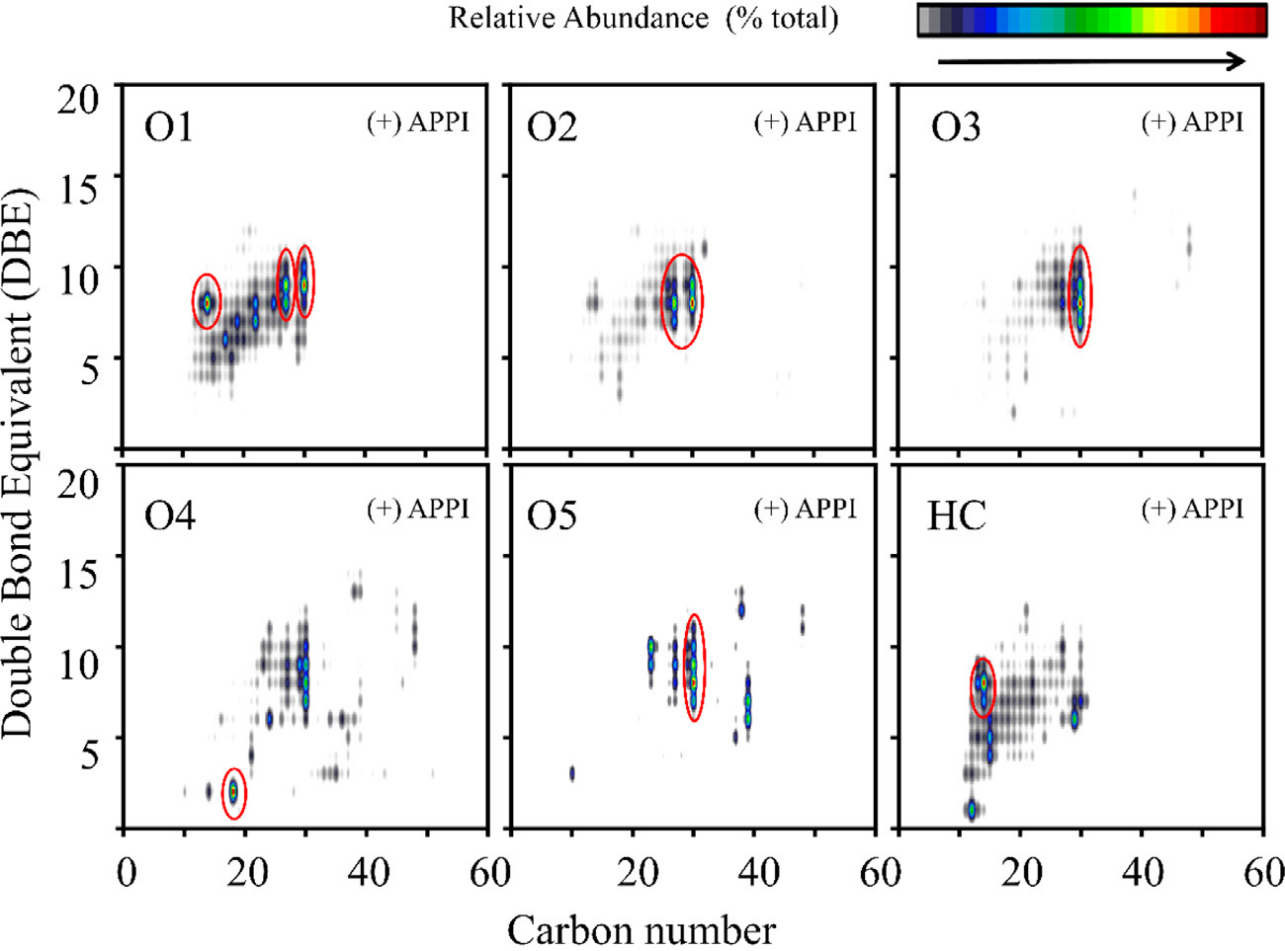
Color-coded isoabundance contour plots for the hydrocarbon (HC) and various heteroatom classes of compounds in guayule resin observed by positive-ion APPI FT-ICR MS.

**Fig. 4:**
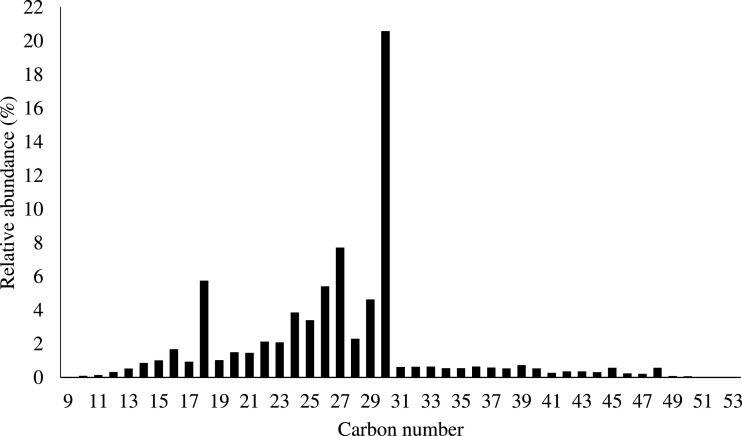
Carbon number distribution in guayule resin from negative-ion APPI FT-ICR MS.

## 2 Experimental design, materials, and methods

Guayule resin from pilot-scale bulk (solvent) rubber extraction was acquired from the Bridgestone Americas Biorubber Processing Research Center (Mesa, AZ), and characterized as received. The guayule plants were harvested at 24–36 months old, field dried for 1–7 days (varies seasonally) to 10–15% moisture, and milled to pass a ¼ in. (6.4 mm) screen. A miscella of rubber and resin was extracted from the whole ground guayule using a mixture of acetone and hexane. Rubber was precipitated from the miscella with addition of excess acetone. Resin was concentrated by evaporation of the solvent from the miscella; after cooling to room temperature, the resin was nearly solid. The resin was collected into barrels, and stored at ambient temperature and humidity (<36% annually) for up to two weeks prior to shipping for analysis. Upon receiving, resin samples were stored in plastic containers at room temperature.

## 3 Gas chromatography mass spectroscopy (MS)

Guayule resin was analyzed by GC–MS to characterize terpene composition. A 10% (w/v) solution was prepared by dissolving resin sample in carbon disulfide. The analyses were performed using a GC–MS system (7890A, Agilent Technologies) equipped with a DB-5 MS capillary column (30 m × 0.25 mm, 0.25 mm, Agilent Technologies). The injection volume of 1 µL was made in a splitless mode. Helium was used as the carrier gas at a flow-rate of 1 mL/min. Oven conditions were initial temperature 50 °C, 1 min isothermal, 3 °C /min ramp to 320 °C, 10 min isothermal. MS conditions included an EI ion source temperature of 250 °C, an ionization energy of 70 eV, and a mass scan range of 50–550 amu. For the identification of terpene compounds, GC–MS libraries including ADAMS and NIST MS Search 2.0 were used, along with a comparison of the linear retention indices with those reported in Adams library [2].

## 4 High-resolution Fourier transform ion cyclotron resonance mass spectroscopy (FT-ICR MS)

Fourier transform ion cyclotron resonance mass spectroscopy (FT-ICR MS) is currently the only analytical technique providing the required resolving power (m/Δm_50%_  ≥ 400,000) and mass accuracy (ppm) for detection and identification of thousands of compounds within a single mass spectrum. This technique typically used to analyze complex natural organic mixtures such as petroleum, biofuels, dissolved organic matter, lipids, and proteins [3]. Guayule resin was analyzed with a custom-built 9.4 T FT-ICR MS at the National High Magnetic Field Laboratory. Atmospheric pressure photoionization (APPI) was used to ionize both polar and non-polar compounds, especially aromatic species, for detection by mass spectrometry. Guayule resin was dissolved in toluene (HPLC grade, JT Baker, Phillipsburg, NJ) to create 1 mg/mL stock solutions. Stock solutions were diluted to a final sample concentration of 10 μg/mL in toluene for positive- and negative-ion atmospheric pressure photoionization. Samples were introduced to the source through a capillary at a rate of 50 μL/min. Nitrogen was used as a sheath gas (60 psi) and auxiliary gas (4 L/min). Inside the heated vaporizer of the source (∼300 °C), the sample was mixed with a nebulization gas (N_2_) and is passed under a krypton VUV lamp producing 10 eV photons (120 nm). Toluene was used to increase ionization efficiency through dopant-assisted photoionization.

Ions generated at atmospheric pressure were introduced into the mass spectrometer via a heated metal capillary. Ions were guided through the skimmer region and quadrupole (mass transfer mode) for accumulation in the second quadrupole. Finally, ions were collisionally cooled with helium gas (∼4 − 5 × 10−6 Torr at gauge) before optimized passage [4] through a transfer quadrupole to the ICR cell. Multiple (50) individual time-domain transients were coadded, Hanning-apodized, zero-filled, and fast-Fourier-transformed prior to frequency conversion to mass-to-charge ratio [5] to obtain the final mass spectrum. The time domain signal acquisition period was 4.1 s. The obtained FT-MS spectrum contained approximately 7200 and 3500 peaks in negative and positive ionization mode respectively, in the m/z range of 150–800.

Data collection was facilitated by a modular ICR data acquisition system (PREDATOR) [6]. Mass spectral lists were generated with PetroOrg software [7]. Internal calibration of the spectrum was based on homologous series whose elemental compositions differ by integer multiples of 14.01565 Da (i.e.,CH_2_) [8, 9]. Data are visualized by relative abundance histograms for heteroatom classes with >1% relative abundance, and from isoabundance-contoured plots of double bond equivalents (DBE = number of rings + double bonds to carbon) versus carbon number for members of a single heteroatom class. The relative abundance scale in isoabundance-contoured plots is scaled relative to the most abundant species in the mass spectrum.

## Declaration of Competing Interest

The authors declare that they have no known competing financial interests or personal relationships that could have appeared to influence the work reported in this paper.
